# Presenting features of female collegiate sports-related concussion in South Africa: a descriptive analysis

**DOI:** 10.17159/2078-516X/2021/v33i1a10416

**Published:** 2021-06-14

**Authors:** R van Tonder, L Kunorozva, PL Viviers, EW Derman, JC Brown

**Affiliations:** 1 Institute of Sport and Exercise Medicine, Division of Orthopaedic Surgery, Faculty of Medicine and Health Sciences, Stellenbosch University, Stellenbosch, South Africa; 2 Campus Health Service, Stellenbosch University, Stellenbosch, South Africa; 3 IOC Research Centre, South Africa

**Keywords:** SRC, depression, anxiety, athlete, outcome

## Abstract

**Background:**

Sports-related concussion (SRC) is an injury with important implications, especially in collision and contact sports, and has a high symptom burden. Student athletes face particular psychosocial challenges, especially female students with pre-existing anxiety/depression are at increased risk for SRC, and have a higher symptom burden before and after injury.

**Objectives:**

Describing female SRC presenting features at a collegiate campus-based sports medicine service; examining the association of prior concussion history (PCONC) and pre-existing anxiety/depression (PMHDx) with SRC.

**Methods:**

A retrospective cohort and statistical analysis (including corrected effect sizes) of Sport Concussion Assessment Tool (versions 3/5) data (Step 1: PCONC and PMHDx history; Step 2: symptom evaluation) of collegiate female athletes with SRC between 2012 and 2018.

**Results:**

Forty females with SRC were identified (age 23 ± 3). The five most frequent symptoms were headache (n = 34; 85%), feeling slowed down (n = 33; 83%), pressure in head (n = 33; 83%), don’t feel right (n = 32; 80%) and fatigue/low-energy (n = 32; 80%). These five symptoms also had the highest self-rated severity (median (IQR): headache (3 (2–4)), feeling slowed down (3 (1–4)), fatigue/low-energy (3 (1–5)), don’t feel right (3 (1–4)) and pressure in head (3 (2–4)). PMHDx (n = 8; 62.9 vs 38.6; p = 0.0192; Hedges’ g_s_ = 0.95; large ES), and not PCONC (n = 13; 51.0 vs 39.8; p = 0.2183; Hedges’ g_s_ = 0.48; small ES) was associated with increased mean total symptom severity.

**Conclusion:**

Headache, feeling slowed down, pressure in head, don’t feel right and fatigue/low-energy had the highest symptom burden. Total symptom severity was no different in those with and without PCONC, but significantly higher in those with PMHDx.

Sports-related concussion (SRC) is defined as a traumatic sports-related brain injury induced by biomechanical forces, caused either by a direct blow to the head, face, neck or elsewhere on the body, with an impulsive force transmitted to the head.^[1]^ Symptomatology (e.g. somatic, cognitive and/or emotional symptoms, physical signs, balance impairment, behavioural changes, cognitive impairment, and sleep/wake disturbances) generally develops predictably but can vary in number, type, severity and time course, and is not due to any other cause (i.e. drugs, alcohol, medication, etc.).^[1]^

Females face a greater SRC risk, sustain SRC at an increased rate, have increased time loss, report more symptoms at baseline and in the immediate post-concussion period, and experience greater neurocognitive impairment. In addition, females have a higher risk for post-concussion syndrome, suffer prolonged recovery from SRC, have an increased risk for depression, and may report a higher number of SRC-related symptoms one year after an SRC.^[2–4]^ Thus, the symptom burden of SRC in female collegiate athletes appears to be high. Furthermore, Wilmoth et al.^[5]^ suggest that student athletes experience several psychological challenges. These challenges may include emotional and social functioning, behavioural problems, academic difficulties, sleep disturbances, headaches and reduced quality of life following SRC, given the importance of athletics and related social activities in their lives. Students have also been shown to experience increased levels of anxiety and depression.^[6]^

Evidence regarding SRC assessment in females is scarce, compared to SRC assessment in males. More data are needed, specifically evaluating females independently,^[7]^ to expand the current evidence base and determine whether females with SRC are presently assessed and managed appropriately.^[3]^ To our knowledge, the association between potential pre-existing risks and SRC has not been investigated to specific symptom type in female collegiate athletes in a culturally diverse developing nation, as the majority of the research in this field has been conducted in developed Northern hemisphere settings. Therefore, we aimed to describe presenting features of female SRC at a collegiate campus-based sports medicine service and investigated the association of presenting symptoms with prior concussion history (PCONC) or mental health conditions (PMHDx).

## Methods

A retrospective cohort analysis of female collegiate student athletes with SRC presenting to a campus-based sports medicine service between the years of 2012 and 2018 was performed. The Stellenbosch University Health Research Ethics Committee granted ethical approval for this study (HREC ref no. N17/08/068).

Electronic medical records were examined for ICD-10 concussion codes (S06.0, S06.00 and S06.01). Only female records were included and analysed for study inclusion eligibility, i.e. concussion cause was classified as sports-related or non-sports-related. Only SRCs were included in this study. The relevant clinical assessment data were extracted from this data subset.

The campus-based sports medicine service is the primary medical care provider for students at the university, which is situated centrally in this university town and therefore highly accessible. The university subsidises all costs of services rendered by this sports medicine service to athletes of the university’s high performance programme. In contrast, non-high performance athletes included in this study were required to cover the cost of services rendered by doctors, either personally or through private medical insurance. The medical service fees were structured to allow for maximum provision of services to students and not to act as a barrier to its use. Furthermore, students could consult, free of charge, with primary nurse practitioners.

All SRCs were diagnosed by an experienced, consistent group of sports physicians at a specialised sports medicine service based on the most recent consensus clinical definitions and guidelines for concussion diagnosis. The SRC assessment included the use of SCAT (versions 3/5, using the currently available version at the time of assessment) questionnaires for all suspected or confirmed concussion cases at initial presentation and subsequent follow-up visits. PCONC and PMHDx data were collected in the SCAT Step 1. The SCAT Step 2 documents the self-reported severity of 22 symptoms based on a 7-point Likert scale of 0–6 (0 = symptom absent, 6 = highest symptom severity), with a maximum possible total severity score of 132 (6 x 22). Total symptom number and severity were also recorded.

The following three outcomes were described and compared between the relevant groups i.e. (1) those with and without PCONC, and (2) those with and without PMHDx: (1) reported severity (0–6) of each of the 22 specific symptoms (e.g. headache, nausea/vomiting, etc.); (2) overall number of reported symptoms (0–22); and (3) overall severity of all reported symptoms (0–132).

Descriptive statistical analyses were performed on all relevant reported data from SCAT Steps 1 and 2. Shapiro-Wilks tests analysed normality, followed by null hypothesis testing (Mann-Whitney U test for non-normal data; parametric unpaired t test for normally distributed data). In the absence of normative reference tables for SCAT test scores, clinically relevant findings for between-group comparisons of the three stated outcomes were determined with the combination of: (1) large (≥0.8) corrected effect size (ES) and (2) appropriate (parametric or non-parametric, depending on data distribution) null hypothesis testing, with a null hypothesis rejected for p < 0.05.

Symptom severity was determined by calculating the mean of all individually reported scores for a specific symptom. Symptom burden was determined as a product of symptom severity and symptom frequency. The two sets of groups that were compared for clinically relevant differences were: 1) between those with and without PCONC and 2) those with and without PMHDx. Statistical comparisons were performed with a combination of Microsoft^®^ Excel for Mac (version 16.37, Microsoft^®^ Corporation) and GraphPad Prism (version 8, GraphPad Software LLC), and verified in Stata (StataCorp. 2011. Stata Statistical Software: Release 12. College Station, TX: StataCorp LP).

## Results

Forty (27% of n = 146) female collegiate athletes with SRC were identified, with a mean age of 23 years (SD ± 3). The included SRC cases were unique, i.e. all patients only had one initial SCAT evaluation. Forty-three records were related to organised sports participation and 103 records were related to recreational, non-sports-related incidents. Three sports-related records had no SCAT data and were therefore excluded from the analysis. Three records related to cycle commuting and one record related to recreational, leisure-time ice skating were deemed to be ‘non-organised’ sports-related causes of concussion for the purpose of this study and excluded from further analysis.^[8]^ Each record noted possible PCONC and PMHDx data. Thirty records (75%) contained data from SCAT3 (2012–2017), and 10 records (25%) contained data from SCAT5 (2017 onwards).

Field hockey (n = 21; 53%) and rugby union (n = 8; 20%) accounted for the highest and second highest number of SRCs, respectively. Other sports involved included cycling (n = 2; 5%), soccer (n = 2; 5%), surfing (n = 2; 5%), netball (n = 2; 5%), water polo (n = 2; 5%), basketball (n = 1; 3%), horse riding (n = 1; 3%), gymnastics (n = 1; 3%) and dancing (n = 1; 3%).

The presenting symptoms are shown in Table 1. Overall, the five most frequent symptoms were headache (n = 34; 85%), feeling slowed down (n = 33; 83%), pressure in head (n = 33; 83%), don’t feel right (n = 32; 80%) and fatigue/low energy (n = 32; 80%). The five most severe symptoms were: headache (median (IQR); 3 (2–4)), feeling slowed down (median (IQR); 3 (1–4)), fatigue/low energy (median (IQR); 3 (1–5)), don’t feel right (median (IQR); 3 (1–4)) and pressure in head (median (IQR); 3 (2–4)). These five symptoms therefore had the highest symptom burden (frequency x severity). The median total symptom number and severity was 13 (IQR 10–17) and 42.5 (IQR 26–62), respectively.

Table 2 summarises data comparing individuals with (n = 8) and without (n = 32) PMHDx. PMHDx was not associated with a higher mean symptom number (15.6 ± 5 vs 12.7 ± 5.7; p = 0.197; moderate ES) but was associated with a significantly higher mean total symptom severity (62.9 ± 9.7 vs 38.6 ± 24.5; p = 0.019; large ES). Headache (p = 0.019; large ES), nausea/vomiting (p = 0.005; large ES), blurred vision (p = 0.036; large ES), sensitivity to light (p = 0.046; large ES), and sensitivity to noise (p = 0.031; large ES) were significantly more severe in those with PMHDx, while the remaining symptoms showed no differences between groups.

When comparing individuals with (n = 13; 32.5%) and without (n = 27; 67.5%) PCONC, we found no differences in individual mean symptom severity, total mean symptom severity or total symptom frequency between groups. Table 3 presents a summary of these data.

## Discussion

The first important finding of this study was that the symptoms most frequently reported overall were headache, feeling slowed down, fatigue/low energy, don’t feel right, and pressure in head - which is consistent with findings in other similar populations.^[2–4, 9, 10]^ The highest mean symptom severity scores were reported for headache, feeling slowed down and fatigue/low energy, don’t feel right, and pressure in head. Combined, this suggests that these five symptoms carry the highest symptom burden in this collegiate female cohort. This finding is consistent with results found in previous research, indicating that females report more somatic symptoms, cognitive difficulties, emotional changes and disordered sleep compared with males.^[3]^ Furthermore, the results from our study supports previous research in collegiate athletes which reported that headache, pressure in head and feeling slowed down were more prevalent in female athletes.^[2]^ A recent study by Vedung et al.^[4]^ in 51 elite Swedish soccer teams during the 2017 season, including 959 elite soccer players (n = 389 females; mean age 23), reported 17 female SRCs and the most frequent post-SRC symptoms were headache, pressure in head, don’t feel right and fatigue/low energy, which are consistent with this study. A subgroup of female semi-professional athletes (aged 23 ± 4; n = 8) in their cohort reported median total symptom severity scores (38.5; IQR 17.75–50.75 vs 42.5; IQR 26–62) and median number of symptoms (14.5; IQR 8.25–18 vs 13; IQR 10–17) - assessed at 48 hours post-SRC - that are also consistent with findings in our study’s cohort. It is important to note that our cohort involved 11 different sports, of which field hockey and rugby union alone accounted for 29 SRCs; in contrast to the Vedung study^[4]^ which focussed solely on soccer players.

This consistency of findings between various research settings, from different geographical areas, various sports, and profoundly different local socio-economic environments,^[2–4, 9, 10]^ suggests that these findings are not unique to our setting and study cohort. Furthermore, South Africa is arguably amongst the most unequal nations globally.^[11]^

Secondly, we found that female collegiate athletes with PMHDx reported significantly higher mean total symptom severity, in addition to a significantly higher severity for physical symptoms such as headache, nausea/vomiting, blurred vision, sensitivity to light and sensitivity to noise, than those athletes without PMHDx. This finding supports previous research findings in similar cohorts of female collegiate athletes.^[3, 12, 13]^ Indeed, Lariviere et al.^[13]^ analysed the electronic records of 4 865 participants, grouped into six age groups (<12 to >40), of whom 1 577 participants self-identified with a diagnosis of anxiety (n = 171 females; aged 16–29 years), depression (n = 119 females; aged 16–29 years), a behavioural disorder, or a learning disability, and reported that pre-existing anxiety or depression worsened SRC symptom numbers and severity. This was in addition to females reporting concussion symptoms with increasing frequency and severity across a large range of age groups, significantly so from the age of 20 years upwards. However, there was no difference in the total symptom number reported between these two groups in our study.

Female sex, previous concussion, and depression were found to be concussion risk factors in a study spanning a period of three years. This study was conducted in a cohort of 10 000 US services academy members (n = 301 concussed females) by Van Pelt et al.^[12]^ The study also reported that females had twice the risk of concussion independent of injury setting, an increased relative risk for SRC, and that the female sex, previous concussion, chronic headache, depression and baseline SCAT scores are significant risk factors for concussion. This study is part of a larger joint effort by the US Department of Defense and National Collegiate Athletic Association that funds the Concussion, Assessment, Research and Education (CARE) Consortium, a multi-site investigation into the natural history of concussion. Cadets at the United States Service Academies are unlike active-duty service members in that there is a larger female population, they have different social, economic, and education characteristics than enlisted service members, and their academy military training activities do not include exposure to combat. Cadets are also required to participate in a sport. Therefore, one could view the service academies as ‘military universities’.

A review by Resch et al.^[3]^ found strong predictive value for prior concussion history, female sex, premorbid psychiatric history and anxiety at follow-up, for post-concussion symptoms one week after sustaining a concussion, in addition to anxiety being predictive of continued symptoms at three months post-concussion. These findings by Resch et al., who reviewed study cohorts, including male and female high school and collegiate athletes, are consistent with findings of a study in a large cohort of > 9 000 youth soccer players by Brooks et al.,^[14]^ which suggested an association between reported post-SRC symptom severity, female sex and prior mental health problems but not with prior concussion history. While the cohort in the study by Brooks et al. consisted of youth athletes, the findings in our study of a female collegiate-level athlete cohort supports the findings by Brooks et al. An additional consideration to be mindful of, as reported in a recent systematic review by Trinh et al.^[7]^, is that pre-existing psychological traits, such as irritability, sadness, nervousness and depressive symptoms, are traits which may predispose an individual to depression and/or anxiety, and were associated with a worsened clinical outcome after SRC. Furthermore, as noted in the study by Lariviere et al., females consistently report higher symptom severity than do males following concussion. Considering that the acute and subacute symptom severity following concussion was shown to be the most consistent predictor of prolonged recovery from concussion,^[15]^ and that individuals with PMHDx were five times more likely to experience prolonged recovery following SRC,^[16]^ it should become clear that the combination of female sex, PMHDx, and generally higher reported post-concussion symptom severity poses a particularly significant clinical management challenge. Therefore, the finding in our study of a significant association between SRC and PMHDx is consistent with current literature and importantly, adds further weight to the existing evidence base.

In contrast to some previous research, we found no association between prior concussion history and the number and severity of reported symptoms in this cohort. Many previous studies have concluded that prior concussion history is a risk factor for future concussion. In a 2014 systematic review by Abrahams et al.^[17]^, which concluded that prior concussion is a risk factor for future concussion, 10 out of 13 reviewed studies found prior concussion history to be a risk factor; however, 9 of these 10 studies included only male cohorts or were 99% male. The remaining 3 (of 13) studies which found otherwise were of a low quality. McCrory et al.^[1]^, Vedung et al.^[4]^, Van Pelt et al.^[12]^, and Putukian et al.^[18]^ also saw prior concussion history to be a risk factor for subsequent SRC.

While this study did not aim to determine concussion risk factors, it is important to note that most of the evidence suggests that PCONC is a risk factor for sustaining a future concussion. In contrast, the evidence regarding clinical outcome following SRC in those with PCONC is equivocal. A comprehensive systematic review of 114 publications by Iverson et al.^[15]^ evaluated various possible outcome predictors following SRC, amongst others sex, PCONC, and prior personal and family psychiatric history. It was found that the majority of studies did not show an association between PCONC, in contrast to female sex and personal/family psychiatric history, and clinical outcome following SRC. The conclusion by this author is consistent with the study by Brooks. et al. (large cohort of >9 000 youth soccer players) that SRC outcome is not associated with PCONC. Our finding supports the absence of an association between PCONC and the clinical outcome following SRC in this small cohort.

With overall increasing rates of sports participation among females, increased concussion injury rates, higher total and more severe reporting of symptoms, and increased risk for prolonged, complicated recovery from SRC among female collegiate athletes, it is imperative that future research continues to expand the body of evidence in this ever-evolving field of SRC, as consideration for differences brought about by biological sex carry important implications for the clinical management of SRC.

From the clinician’s perspective, these findings bear important clinical relevance. The clinician should be mindful thereof in the management of female collegiate athletes who present for baseline testing or post-injury assessment, in particular those with PMHDx and, although the present cohort showed no association with those with prior concussion history.

The data used in this study are based on the information contained in the SCAT3/5 questionnaires which were completed as a central component in the assessment of suspected cases of SRC at this collegiate campus-based sports medicine service, the primary difference (in step 2) being that ‘state’ is reported in SCAT3 compared to ‘trait’ in SCAT5. During the study period, the SCAT5 was released following the Berlin Consensus meeting ^[1]^ and although SCAT3 and SCAT5 questionnaires are described in this study, Asken et al.^[19]^ found adequate convergent validity between SCAT3 and SCAT5. The symptomatology data collected in the SCAT questionnaires rely on the self-reporting of symptoms that is known to introduce bias, as these data cannot be verified independently. The non-parametric nature of these collected SCAT data, which contain large amounts of zero scores, cause additional statistical constraints. Furthermore, it is unclear what the impact of elapsed time is between previous and instant concussions. This requires further study.

Our statistical measures employed, i.e. the addition of an ES measure to differentiate more clearly meaningful clinical findings were strict when compared to previous studies. Furthermore, in our study, which could be viewed as both a strength and limitation, all SRCs were diagnosed by an experienced consistent group of sports physicians at a specialised sports medicine service in accordance with the most recent consensus clinical definitions and guidelines for concussion diagnosis.^[1]^ This could imply a more narrowly defined cohort of diagnosed concussed athletes, in addition to differences in assessment methods and questionnaires employed elsewhere around the world. Recall errors at the time of questionnaire completion may also influence accurate recording of prior concussion history in the athlete who presents with a current concussion.

The data analysis derived from this work could be affected by the small sample size, impacting on certain statistical relationships which the cohort in this study did not show but which have been shown to exist in other studies. However, comparative studies also report small female collegiate SRC cohort sizes.^[13, 18, 20–23]^ The small sample size is in spite of the multiyear time period under consideration in this study. This should not be construed as an indication of overall low female SRC incidence. Indeed, the SRC incidence rate was not a focus of this work. Rather, it may be possible that the SRC surveillance and referral systems which exist in women’s sports codes on the university’s campus do not lead to an accurate portrayal of the true SRC incidence. Furthermore, local socio-economic and cultural factors may influence an athlete’s awareness and understanding of SRC and the ability to seek medical care after sustaining an SRC. Therefore, not all SRC cases may have been recorded. The data analysis was hampered by the fact that three eligible SRC records had missing SCAT data.

## Conclusion

This study aims to answer the call for more female specific SRC research. Our findings suggest that the number and severity of symptoms are consistently reported in similar female collegiate athlete cohorts, in addition to similar symptoms being reported in different study cohorts competing in different sports. Symptoms with the highest burden were headache, feeling slowed down, pressure in head, don’t feel right and fatigue/low energy. We found no difference in the reported number and severity of symptoms between those with and without PCONC. However, total and physical symptom cluster severity was significantly higher in those with PMHDx compared to athletes without PMHDx. The finding of a significant association between PMHDx and the SRC clinical outcome is consistent with previous research. Whereas previous research is equivocal regarding an association between PCONC and SRC outcome, the present cohort showed no such association. We encourage further research of female-specific SRC to expand our knowledge and ultimately improve the health of female athletes.

## Figures and Tables

**Table 1:** Overall (n = 40) mean symptom severity and symptom frequency

	n (%)	Mean ± SD	Median (IQR)
**Number of symptoms**		13.3 ± 5.6	13 (10–17)
**Symptom severity score**		43.5 ± 26.7	42.5 (26–62)
**Headache**	34 (85)	3.0 ± 1.7	3 (2–4)
**Pressure in head**	33 (83)	2.8 ± 1.8	3 (2–4)
**Neck pain**	27 (68)	2.1 ± 1.9	2 (0–4)
**Nausea/vomiting**	21 (53)	1.7 ± 1.9	1 (0–3)
**Dizziness**	31 (78)	1.9 ± 1.5	2 (1–3)
**Blurred vision**	15 (38)	0.9 ± 1.4	0 (0–1)
**Balance problems**	16 (40)	1.1 ± 1.6	0 (0–2)
**Sensitivity to light**	23 (58)	1.6 ± 1.7	1 (0–3)
**Sensitivity to noise**	19 (48)	1.5 ± 1.9	0 (0–3)
**Feeling slowed down**	33 (83)	2.9 ± 1.9	3 (1–4)
**Feeling like in a fog**	27 (68)	2.4 ± 2.0	3 (0–4)
**Don** **’** **t feel right**	32 (80)	2.8 ± 1.9	3 (1–4)
**Difficult concentrating**	28 (70)	2.5 ± 2.2	2 (0–4)
**Difficulty remembering**	24 (60)	1.8 ± 1.8	2 (0–3)
**Fatigue/low energy**	32 (80)	2.9 ± 2.2	3 (1–5)
**Confusion**	19 (48)	1.5 ± 1.9	0 (0–2)
**Drowsiness**	28 (70)	2.4 ± 2.0	2 (0–4)
**Trouble falling asleep**	18 (45)	1.8 ± 2.2	0 (0–3)
**More emotional**	20 (50)	1.8 ± 2.1	1 (0–4)
**Irritability**	19 (48)	1.8 ± 2.1	0 (0–4)
**Sadness**	17 (43)	1.3 ± 1.8	0 (0–2)
**Nervous/anxious**	17 (43)	1.2 ± 1.7	0 (0–2)

n, absolute frequency of symptoms; %, percentage of symptom frequency; SD, standard deviation; IQR, interquartile range

**Table 2:** No prior mental health diagnosis vs prior mental health diagnosis (PMHDx) - symptom severity and symptom frequency

	No PMHDx (n = 32)			PMHDx (n = 8)									

	n (%)	Mean ± SD	Median (IQR)	n (%)	Mean ± SD	Median (IQR)	MΔ	MΔ %	d_s_	g_s_	ES	U	t

**Number of symptoms**		12.7 ± 5.7	13 (10–17)		15.6 ± 5.0	16 (9–20)	2.9	22.9	0.52	0.51	**		0.197^
**Symptom severity score**		38.6 ± 24.5	37 (21–58)		62.9 ± 27.5	65 (29–86)	24.3	62.9	0.97	0.95	***		0.019^
**Headache**	26 (81)	2.7 ± 1.7	3 (2–4)	8 (100)	4.1 ± 0.8	4 (3–5)	1.5	55.3	0.92	0.91	***	0.019^	
**Pressure in head**	25 (78)	2.6 ± 1.8	3 (2–4)	8 (100)	3.4 ± 1.6	3 (1–4)	0.8	28.6	0.43	0.42	*	0.354	
**Neck pain**	23 (72)	2.1 ± 1.8	2 (0–4)	4 (50)	1.9 ± 2.2	1 (0–4)	−0.3	−11.8	−0.13	−0.13	*	0.689	
**Nausea/Vomiting**	14 (44)	1.2 ± 1.7	0 (0–2)	7 (88)	3.4 ± 1.9	4 (0–4)	2.2	176.9	1.25	1.22	***	0.005^	
**Dizziness**	24 (75)	1.7 ± 1.4	2 (1–3)	7 (88)	2.8 ± 1.7	3 (0–4)	1	60	0.7	0.69	**	0.113	
**Blurred vision**	10 (31)	0.6 ± 1.1	0 (0–1)	5 (63)	2.0 ± 2.0	2 (0–3)	1.4	255.6	1.11	1.09	***	0.036^	
**Balance problems**	12 (38)	0.9 ± 1.4	0 (0–2)	4 (50)	1.9 ± 2.3	1 (0–3)	0.9	100.4	0.58	0.57	**	0.309	
**Sensitivity to light**	17 (53)	1.2 ± 1.4	1 (0–2)	6 (75)	2.9 ± 2.2	3 (0–4)	1.7	135.9	1.04	1.02	***	0.046^	
**Sensitivity to noise**	13 (41)	1.2 ± 1.7	0 (0–2)	6 (75)	2.9 ± 2.2	3 (0–4)	1.7	148.6	0.95	0.94	***	0.031^	
**Feeling slowed down**	25 (78)	2.7 ± 1.9	3 (1–4)	8 (100)	4.0 ± 1.1	4 (3–4)	1.3	50.6	0.75	0.73	**	0.1	
**Feeling like in a fog**	21 (66)	2.2 ± 1.9	3 (0–4)	6 (75)	3.0 ± 2.5	4 (0–5)	0.8	35.2	0.39	0.38	*	0.37	
**Don** **’** **t feel right**	24 (75)	2.5 ± 1.9	3 (1–4)	8 (100)	3.9 ± 1.6	4 (1–5)	1.3	53.1	0.72	0.71	**	0.075	
**Difficulty concentrating**	21 (66)	2.3 ± 2.2	2 (0–4)	7 (88)	3.3 ± 1.9	4 (0–4)	1	44.4	0.47	0.46	*	0.231	
**Difficulty remembering**	18 (56)	1.6 ± 1.7	2 (0–3)	6 (75)	2.6 ± 2.0	3 (0–4)	1	64.7	0.58	0.57	**	0.17	
**Fatigue/low energy**	26 (81)	2.7 ± 2.1	3 (1–4)	6 (75)	3.5 ± 2.3	4 (0–5)	0.8	28.7	0.36	0.35	*	0.411	
**Confusion**	14 (44)	1.2 ± 1.7	0 (0–2)	5 (63)	2.6 ± 2.5	3 (0–4)	1.5	127	0.8	0.78	**	0.113	
**Drowsiness**	22 (69)	2.1 ± 1.9	2 (0–3)	6 (75)	3.6 ± 2.3	5 (0–5)	1.5	73.1	0.78	0.77	**	0.059	
**Trouble falling asleep**	15 (47)	1.7 ± 2.1	0 (0–3)	3 (38)	1.9 ± 2.8	0 (0–4)	0.2	9.1	0.07	0.07	*	0.989	
**More emotional**	16 (50)	1.6 ± 2.0	1 (0–4)	4 (50)	2.4 ± 2.6	2 (0–4)	0.8	46.2	0.36	0.35	*	0.506	
**Irritability**	15 (47)	1.6 ± 1.9	0 (0–3)	4 (50)	2.5 ± 2.7	2 (0–5)	0.9	60	0.45	0.44	*	0.357	
**Sadness**	14 (44)	1.2 ± 1.5	0 (0–2)	3 (38)	1.9 ± 2.6	0 (0–4)	0.7	57.9	0.38	0.38	*	0.708	
**Nervous/anxious**	13 (41)	1.00 ± 1.4	0 (0–2)	4 (50)	2.1 ± 2.5	2 (0–4)	1.1	112.5	0.68	0.66	**	0.286	

n, absolute frequency of symptoms; %, percentage of symptom frequency; SD, standard deviation; IQR, interquartile range; M**Δ**, mean difference; M**Δ** %, mean difference percentage; d_s_, Cohen’s d_s_; g_s_, Hedges’ g_s_; ES, effect size; U, Mann Whitney U test; t, Unpaired t test;

^ ,p < 0.05;

* , small ES;

** , moderate ES;

*** , large ES.

**Table 3:** No prior concussion vs prior concussion (PCONC) - symptom severity and symptom frequency

	No PCONC (n = 27)			PCONC (n = 13)									

	n (%)	Mean ± SD	Median (IQR)	n (%)	Mean ± SD	Median (IQR)	MΔ	MΔ %	d_s_	g_s_	ES	*U*	*t*

**Number of symptoms**		12.7 ± 6.1	13 (10–17)		14.5 ± 4.6	15 (11–18)	1.7	13.5	0.3	0.30	*		0,373
**Symptom severity score**		39.8 ± 25.6	37 (25–60)		51.0 ± 28.4	46 (32–81)	11.2	28.1	0.49	0.48	*		0,218
**Headache**	21 (78)	2.7 ± 1.8	3 (2–4)	8 (100)	3.5 ± 1.2	4 (3–4)	0.9	32.7	0.53	0.52	**	0,146	
**Pressure in head**	21 (78)	2.6 ± 1.7	3 (2–4)	8 (92)	3.2 ± 1.8	3 (2–5)	0.6	21.6	0.32	0.31	*	0,370	
**Neck pain**	19 (70)	2.0 ± 1.9	1 (0–3)	4 (62)	2.3 ± 2.0	3 (0–4)	0.3	17.6	0.18	0.18	*	0,655	
**Nausea/vomiting**	13 (48)	1.4 ± 1.8	0 (0–2)	7 (62)	2.2 ± 2.2	2 (0–4)	0.7	53	0.39	0.38	*	0,315	
**Dizziness**	20 (74)	1.6 ± 1.4	1 (1–3)	7 (85)	2.6 ± 1.6	3 (2–4)	1	64.2	0.71	0.69	**	0,055	
**Blurred vision**	10 (37)	0.7 ± 1.1	0 (0–1)	5 (38)	1.2 ± 1.8	0 (0–3)	0.6	84.6	0.4	0.4	*	0,603	
**Balance problems**	9 (33)	0.8 ± 1.3	0 (0–1)	4 (54)	1.8 ± 2.1	1 (0–3)	1	127.5	0.63	0.61	**	0,134	
**Sensitivity to light**	14 (52)	1.2 ± 1.4	1 (0–2)	6 (69)	2.4 ± 2.1	3 (0–4)	1.2	107.7	0.76	0.75	**	0,071	
**Sensitivity to noise**	11 (41)	1.2 ± 1.8	0 (0–3)	6 (62)	2.1 ± 2.1	2 (0–4)	0.9	69.9	0.45	0.44	*	0,160	
**Feeling slowed down**	22 (81)	2.8 ± 1.9	3 (1–4)	8 (85)	3.2 ± 1.9	3 (3–4)	0.3	12	0.18	0.18	*	0,630	
**Feeling like in a fog**	19 (70)	2.4 ± 1.9	3 (0–4)	6 (62)	2.3 ± 2.4	1 (0–4)	−0.1	−4.1	−0.05	−0.05	*	0,926	
**Don** **’** **t feel right**	20 (74)	2.6 ± 2.0	3 (1–4)	8 (92)	3.2 ± 1.8	4 (2–4)	0.5	19.9	0.27	0.27	*	0,406	
**Difficult concentrating**	17 (63)	2.2 ± 2.2	2 (0–4)	7 (85)	2.9 ± 2.0	3 (2–4)	0.7	31.5	0.33	0.32	*	0,312	
**Difficulty remembering**	15 (56)	1.6 ± 1.7	1 (0–3)	6 (69)	2.2 ± 2.0	2 (0–3)	0.6	40.1	0.35	0.35	*	0,345	
**Fatigue/low energy**	22 (81)	2.9 ± 2.1	3 (1–5)	6 (77)	2.9 ± 2.4	4 (1–5)	0	−1.5	−0.02	−0.02	*	1,000	
**Confusion**	13 (48)	1.3 ± 1.6	0 (0–2)	5 (46)	1.9 ± 2.5	0 (0–4)	0.6	46.6	0.31	0.30	*	0,681	
**Drowsiness**	19 (70)	2.3 ± 1.9	2 (0–4)	6 (69)	2.7 ± 2.3	4 (0–4)	0.4	19.2	0.21	0.21	*	0,539	
**Trouble falling asleep**	13 (48)	1.9 ± 2.3	0 (0–4)	3 (38)	1.4 ± 2.0	0 (0–3)	−0.5	−28.1	−0.25	−0.24	*	0,419	
**More emotional**	13 (48)	1.7 ± 2.1	0 (0–4)	4 (54)	2.0 ± 2.2	1 (0–4)	0.3	20	0.16	0.16	*	0,708	
**Irritability**	11 (41)	1.5 ± 2.1	0 (0–3)	4 (62)	2.2 ± 2.1	2 (0–4)	0.7	46.9	0.34	0.33	*	0,231	
**Sadness**	12 (44)	1.3 ± 1.7	0 (0–2)	3 (38)	1.4 ± 2.1	0 (0–3)	0.1	6.8	0.05	0.05	*	0,954	
**Nervous/anxious**	11 (41)	1.2 ± 1.7	0 (0–2)	4 (46)	1.4 ± 1.8	0 (0–3)	0.2	20.6	0.14	0.13	*	0,678	

n, absolute frequency of symptoms; %, percentage of symptom frequency; SD, standard deviation; IQR, interquartile range; M**Δ**, mean difference; M**Δ** %, mean difference percentage; d_s_, Cohen’s d_s_; g_s_, Hedges’ g_s_; ES, effect size; U, Mann Whitney U test; t, Unpaired t test;

* , small ES;

** , moderate ES;

*** , large ES.
